# Sex differences in the non-linear association between BMI and LDL cholesterol in middle-aged and older adults: findings from two nationally representative surveys in China

**DOI:** 10.1186/s12944-021-01591-w

**Published:** 2021-11-14

**Authors:** Haibin Li, Jiahui Ma, Deqiang Zheng, Xia Li, Xiuhua Guo, Jing Wang, Pixiong Su

**Affiliations:** 1 Department of Cardiac Surgery, Heart Center, Beijing Chaoyang Hospital, Capital Medical University, Beijing, China; 2 Beijing Key Laboratory of Hypertension, Beijing Chaoyang Hospital, Capital Medical University, Beijing, China; 3 Department of Anesthesiology and Critical Care Medicine, Peking University First Hospital, Beijing, China; 4 Department of Epidemiology and Health Statistics, School of Public Health, Capital Medical University, Beijing, China; 5 Department of Mathematics and Statistics, La Trobe University, Melbourne, Victoria Australia; 6 Department of Clinical Laboratory, Beijing Chaoyang Hospital, Capital Medical University, Beijing, China

**Keywords:** BMI, LDL-C, Nonlinear relationship, Sex differences, Generalized additive models

## Abstract

**Background:**

The relationship between body mass index (BMI) and low-density lipoprotein cholesterol (LDL-C) has not been clearly elucidated in middle-aged and older adults. This study aimed to evaluate the non-linear dose-response relationship between BMI and LDL-C in males and females.

**Methods:**

Data was obtained from two nationally representative surveys in China—the China Health and Nutrition Survey (CHNS, 2009) and China Health and Retirement Longitudinal Study (CHARLS, 2011–2012). To evaluate the sex differences in the association between BMI and LDL-C, the generalized additive models with a smooth function for continuous BMI and smooth-factor interaction for sexes with BMI were used. Segmented regressions were fitted to calculate the slopes with different estimated breakpoints among females and males.

**Results:**

A total of 12,273 participants (47.1% male) aged 45 to 75 years were included. The generalized additive models revealed that a non-linear relationship between BMI and LDL-C level in both sexes after adjustment for age, residence, education levels, marital status, drinking, smoking status, and cohort (CHNS or CHARLS). Slopes of the association between BMI and LDL-C association changed at BMI 20.3 kg/m^2^ (95% CI: 18.8 to 21.8) in females and 27.1 kg/m^2^ (95% CI: 25. 8 to 28.4) in males. Below these BMI breakpoints, LDL-C levels increased 1.84 (95% CI: 1.45 to 2.31) in males and 3.49 (95% CI: 1.54 to 5.45) mg/dL per kg/m^2^ in females. However, LDL-C levels declined − 1.50 (95% CI: − 2.92 to − 0.09) mg/dL per kg/m^2^ above BMI of 27.1 kg/m^2^ in males. The non-linear association BMI and LDL-C in males and females was varied by cohort source, age groups, and the number of metabolic syndrome criteria.

**Conclusions:**

In the Chinese middle aged and older adults, the BMI and LDL-C relationship was inverted U-shaped with a high level of LDL-C at a BMI of 27.1 kg/m^2^ in males, and an approximately linear association was observed in females.

**Supplementary Information:**

The online version contains supplementary material available at 10.1186/s12944-021-01591-w.

## Introduction

Globally, higher body mass index (BMI), a reliable overweight and obesity marker, has been a serious public health concern. It is postulated that by 2030, the prevalence of overweight and obesity will be 23 and 32%, respectively [1]. Overweight and obesity affect individuals of all ages, but especially common among the middle-aged adults. High BMI is associated with the occurrence of cardiovascular diseases (CVD) [2, 3]. It has been reported that for every 5 kg/m^2^ increased in BMI, the average of all-cause mortality and vascular mortality increases by 30 and 40%, respectively [4].

As one of the causes of CVD, metabolic syndrome (MS) is associated with higher BMI and dyslipidemia, which are due to elevated triglycerides (TG) and reduced high-density lipoprotein cholesterol (HDL-C), but without a clear effect on low-density lipoprotein cholesterol (LDL-C) [5]. However, Mendelian randomization and epidemiological studies have suggested that LDL-C is a risk factor for CVD [6–8]. Moreover, compared to HDL-C and TG, the concentration of LDL-C and its therapeutic modification are greatly associated with CVD [9–11].

Previous studies evaluated the linear relationship between LDL-C and BMI in limited or selected samples, however, data on potential sex differences is limited [12–14]. For example, a study performed in the US population suggested that LDL-C linearly associated with BMI [12]. Another cohort study involving US children also indicated that BMI is a strong determinants of lipid and lipoprotein levels [13]. One study reported that LDL-C only increased with greater BMI among younger subjects, but not among male over 50 years [14]. Notably, a recent large population-based study involving individuals form both US and Spain found that there is not always a linear relationship between LDL-C and BMI, that is, age and metabolic status play essential roles and effects on relationship [15]. However, although a visceral fat area was found to be positively correlated with serum LDL-C levels in a non-diabetic Chinese population [16], associations between BMI and LDL-C are yet to be clearly defined. Moreover, since fat distribution differs between the sexes, the effects of sex differences on the above associations should be determined in other ethnicity population.

Therefore, this study was aimed to investigate the non-linear dose-response relationship between BMI and LDL-C and determined whether there are sex differences in a Chinese adult population. We hypothesized that LDL-C levels increased with BMI only in lean individuals and sex-related differences were existed. Our findings will inform on the prevention and treatment of CVD in Asian populations.

## Methods

### Study participants

This study analyzed two cross-sectional nationally representative datasets from the China Health and Nutrition Survey (CHNS) and the China Health and Retirement Longitudinal Study (CHARLS). Detailed descriptions of these two studies have been published [17, 18]. Briefly, the CHNS is an ongoing open cohort study designed to establish the association between socio-economic factors and health change in China [17]. The CHNS was initially conducted in 1989, while the biomarker data were first collected in 2009. The CHARLS, a nationally representative longitudinal study involving 17,708 adults in 150 counties of 28 provinces in China, was conducted in 2011–12. This study has information on demographic characteristics, medical history, lifestyles, and laboratory data for a set of individuals [18].

The CHARLS was approved by the Biomedical Ethics Review Committee of Peking University, and the CHNS were approved by the Institutional Review Boards at the University of North Carolina at Chapel Hill, the Institute of Nutrition and Food Safety, Chinese Center for Disease Control and Prevention. All participants from CHNS and CHARLS provided written informed consents.

**Fig. 1:**
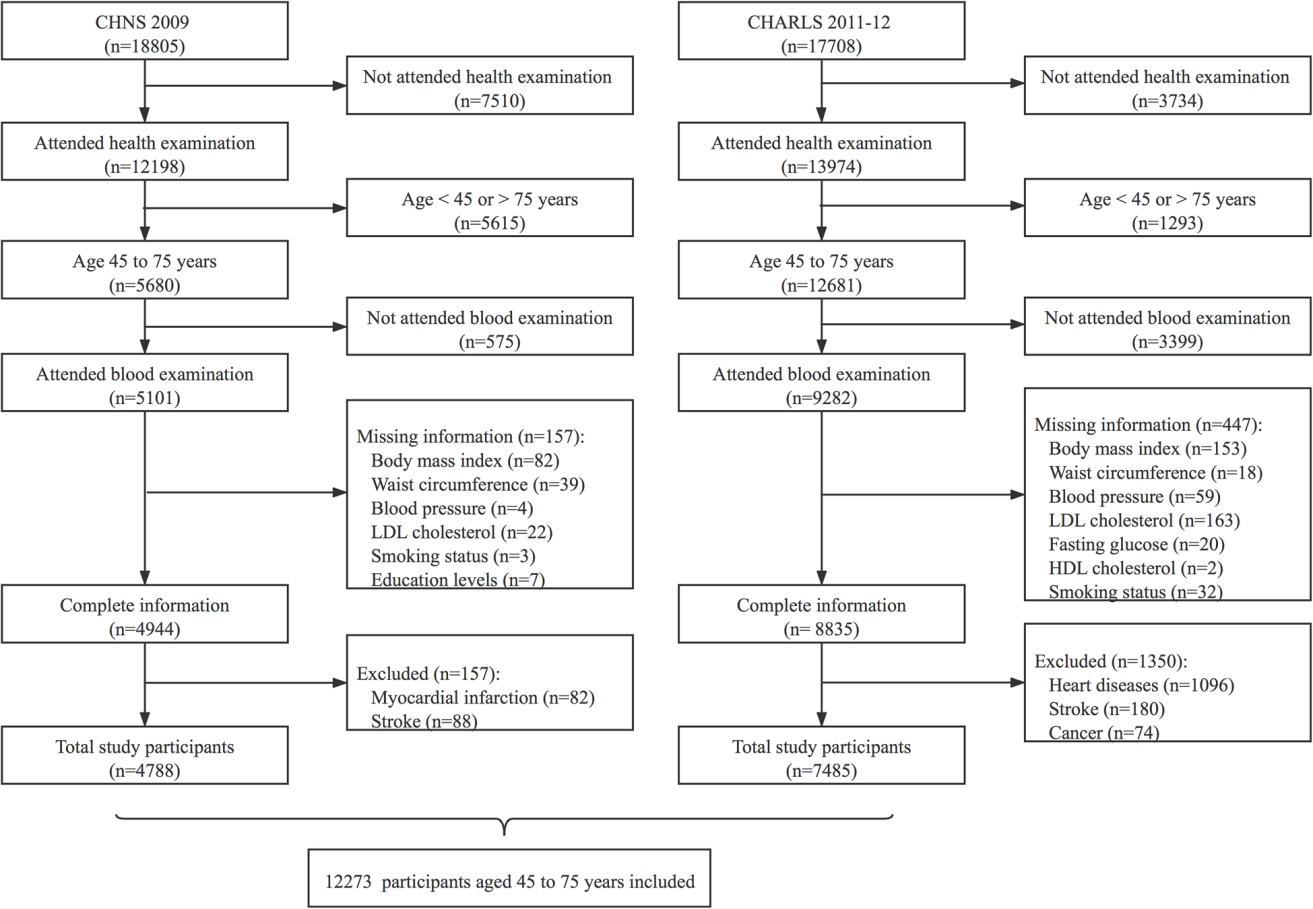
Flow chart of participant selection

In this study, adults aged between 45 and 75 years whose data on blood biomarkers and anthropometric measures were available in both datasets were identified. Participants with missing baseline covariates or those with CVD or cancer were excluded. Finally, a total of 12,273 participants (*n* = 4788 from CHNS; *n* = 7485 from CHARLS) were included (Fig. 1).

### Data collection

Data on age, sex, residence (urban or rural), education level (< high school or ≥ high school), marital status (married or others), current smoking and drinking status (yes or no), menopause status (yes or no [only available in CHALRS]) and medical history (including hypertension, diabetes, heart disease, stroke, cancer) and drug use (including hypertension medications, diabetes medications, lipid-lowering therapy [only available in CHALRS]) were obtained from the standard questionnaires and harmonized for both datasets. Blood samples were obtained and measured for serum lipids [total cholesterol (TC), TG, LDL-C, HDL-C] and blood glucose. In each survey, height, bodyweight, waist circumference (WC) and blood pressure were measured by trained investigators, according to a standardized protocol. BMI was calculated as weight in kilograms divided by the square of height in meters and categorized into underweight (< 18.5 kg/m^2^), normal (18.5–23.9 kg/m^2^), overweight (24.0–27.9 kg/m^2^), and obese (≥ 28.0 kg/m^2^). Hypertension was defined as self-reported history of hypertension, systolic blood pressure ≥ 140 mmHg, diastolic blood pressure ≥ 90 mmHg, or use of anti-hypertensive drugs. Diabetes was defined as self-reported history of diabetes, fasting blood glucose ≥126 mg/dl, or as the use of anti-diabetic therapy. Hypercholesterolemia was defined as total cholesterol >240 mg/dl or use of lipid-lowering therapy. MS was defined based on the definition in “Harmonizing the metabolic syndrome 2009” which included: elevated WC (≥ 85 cm in male, ≥ 80 cm in female); elevated TG (≥ 150 mg dl); low HDL-C (< 40 mg/dl in male and < 50 mg/dl in female); elevated blood pressure (systolic ≥130 and/or diastolic ≥85 mmHg) or current treatment for hypertension; and elevated fasting glucose (fasting glucose ≥100 mg/dl or current treatment for diabetes) [19]. Cardiometabolic health was defined as the number of MS components (excluding WC due to a strong correlation between BMI and WC [*r* = 0.64, *P* < 0.001]).

### Statistical analysis

Participant characteristics by sexes were separately described in CHARLS and CHNS. Differences between sexes were assessed using the t-test or Mann-Whitney U test for continuous variables, and Pearson’s χ^2^ test for categorical variables.

To investigate the non-linear association between BMI and LDL-C among females and males, generalized additive models (GAMs) with a factor-smooth interaction between sex (factor) and BMI (smooth function) were fitted. Models were adjusted for age, residence, education levels, marital status, drinking, smoking status, and cohort sources (when pooled analysis). Thus, the multivariable model can be expressed as:

$$ {Y}_i={\beta}_0+\left[{f}_{BMI,}\mathrm{SEX}\left( BM{I}_i\right)\times \mathrm{I}\left({x}_{\mathrm{sex}}=1\right)\right]+\left[{f}_{BMI,}\overline{sex}\left( BM{I}_i\right)\times \mathrm{I}\left({x}_{\mathrm{sex}}=0\right)\right]+{\beta}_{\mathrm{sex}}{x}_{\mathrm{sex}}+\beta {Cov}_i^T+{\varepsilon}_i $$

where *Y*_*i*_ is the response of LDL-C for individual *i*; *f*_*BMI*_ is the nonparametric smooth function of BMI; *β*_sex_*x*_sex_ is the main effect of the sex variable (0 = females, 1 = males); $\beta {Cov}_i^T$ is the linear predictor of covariables, and *ε*_*i*_ is the error terms, which are assumed to be normally distributed. The relationship between BMI and LDL-C was visualized via the GAMs smooth plot by sexes. The association between BMI and other serum lipids (TC, HDL-C, and TG) by sexes was additionally examined as an exploratory analysis.

Then, a multivariable adjusted generalized linear model using linear splines, with break points identified from GAMs smooth plot were separately performed in females and males. Two multivariable models were built: (1) adjusted for age, residence, education levels, marital status, drinking, smoking status, and cohort sources (when pooled analysis); (2) additionally adjusted for hypertension and diabetes. In the CHARLS cohort, use of lipid-lowering (Model 2a) and menopause status in females (Model 2b) were additionally adjusted as a sensitivity analysis.

In addition, to elucidate on age and cardiometabolic heath-based variations in the relationship between BMI and LDL-C in females and males, subgroup analyses were performed stratified by CHNS and CHARLS, age-groups (45–54, 55–64, 65–75 years) and the number of MS criteria (0, 1, 2, 3 or 4 after excluding WC criterion).

 Analyses were performed using Stata 16.0 (Stata Corp LLC) and R statistical software (version 4.0.5), with the “mgcv”, “segmented” and “ggplot2” package. Two-tailed *P* < 0.05 was considered statistically significant.

## Results

### Baseline characteristics

In the current analysis, 12,273 adults (6493 females and 5780 males) aged 45–75 years were included. The characteristics of study population by sexes in CHARLS and CHNS are shown in Table 1. There were significant differences between females and males with regards to BMI, LDL-C, education level, marital status, smoking, drinking, hypercholesterolemia, hypertension medications, diastolic blood pressure, TC, TG, HDL-C, and count of MS criteria in both cohorts (*P* < 0.05). The distribution of BMI and LDL-C in females and males in CHARLS and CHNS are displayed in Supplementary Fig. 1. Baseline characteristics, stratified by CHARLS and CHNS, are shown in Supplementary Table 1.

**Table 1:** Baseline Characteristics by Sex in CHARLS and CHNS

	CHARLS				CHNS			
	All (*n* = 7485)	Female (*n* = 3933)	Male (*n* = 3522)	*P* value	All (*n* = 4788)	Female (*n* = 2560)	Male (*n* = 2228)	*P* value
Age, years, mean (SD)	58 (8)	57 (8)	58 (8)	< 0.001#	57 (8)	57 (8)	57.4 (8)	0.718#
45–54 years	2831 (37.8)	1595 (40.6)	1236 (34.8)		1959 (40.9)	1047 (40.9)	912 (40.9)	
55–64 years	3083 (41.2)	1587 (40.4)	1496 (42.1)		1784 (37.3)	949 (37.1)	835 (37.5)	
65–75 years	1571 (21.0)	751 (19.1)	820 (23.1)		1045 (21.8)	564 (22.0)	481 (21.6)	
Rural residence, n (%)	4924 (65.8)	2566 (65.2)	2358 (66.4)	0.298*	3274 (68.4)	1736 (67.8)	1538 (69.0)	0.366*
High school or above, n (%)	760 (10.2)	276 (7.0)	484 (13.6)	< 0.001*	988 (20.6)	418 (16.3)	570 (25.6)	< 0.001*
Married, n (%)	6405 (85.6)	3239 (82.4)	3166 (89.1)	< 0.001*	4224 (88.2)	2172 (84.8)	2052 (92.1)	< 0.001*
Drinking, n (%)	2617 (35.0)	514 (13.1)	2103 (59.2)	< 0.001*	1585 (33.1)	227 (8.9)	1358 (61.0)	< 0.001*
Smoking, n (%)	2393 (32.0)	211 (5.4)	2182 (61.4)	< 0.001*	1396 (29.2)	129 (5.0)	1267 (56.9)	< 0.001*
BMI, kg/m^2^, mean (SD)	23.4 (3.5)	23.9 (3.6)	22.9 (3.3)	< 0.001#	23.7 (3.3)	23.9 (3.4)	23.5 (3.3)	< 0.001#
Underweight	476 (6.4)	241 (6.1)	235 (6.6)		242 (5.1)	131 (5.1)	111 (5.0)	
Normal	4000 (53.4)	1863 (47.4)	2137 (60.2)		2451 (50.4)	1244 (48.6)	1171 (52.6)	
Overweight	2193 (29.3)	1282 (32.6)	911 (25.6)		1613 (33.7)	864 (33.8)	749 (33.6)	
Obese	816 (10.9)	547 (13.9)	269 (7.6)		518 (10.8)	321 (12.5)	197 (8.8)	
Hypertension, n (%)	2914 (38.9)	1565 (39.8)	1349 (38.0)	0.108*	1859 (38.8)	959 (37.5)	900 (40.4)	0.038*
Diabetes, n (%)	1140 (15.2)	590 (15.0)	550 (15.5)	0.561*	463 (9.7)	219 (8.6)	244 (11.0)	0.005*
Hypercholesterolemia, n (%)	1072 (14.3)	658 (16.7)	414 (11.7)	< 0.001*	577 (12.1)	371 (14.5)	206 (9.2)	< 0.001*
History of medication use, n (%)								
Hypertension medications	1163 (15.5)	656 (16.7)	507 (14.3)	0.004*	184 (3.8)	85 (3.3)	99 (4.4)	0.044*
Diabetes medications	233 (3.1)	131 (3.3)	102 (2.9)	0.253*	169 (3.5)	80 (3.1)	89 (4.0)	0.104*
Lipid-lowering therapy	287 (3.8)	160 (4.1)	127 (3.6)	0.268*	NA	NA	NA	
Menopause, n (%)	2864 (72.8)	2864 (72.8)	NA		NA	NA	NA	
Waist circumference, cm, mean (SD)	83.9 (12.1)	84.2 (12.4)	83.7 (11.8)	0.088#	84.3 (9.9)	83.4 (9.8)	85.3 (10.0)	< 0.001#
Systolic BP, mmHg, mean (SD)	130 (21)	129.4 (21.8)	129.7 (20.1)	0.525#	129 (19)	129 (20)	129 (18)	0.318#
Diastolic BP, mmHg, mean (SD)	76 (12)	75.3 (11.9)	76.3 (12.4)	< 0.001#	82 (11)	82 (11)	84 (11)	< 0.001#
Fasting glucose, mg/dl, mean (SD)	109.5 (36.2)	109.3 (36.9)	109.7 (35.4)	0.615#	100.0 (28.6)	99.0 (25.9)	101.2 (31.4)	0.009#
Total cholesterol, mg/dl, mean (SD)	193.2 (37.9)	197.8 (37.9)	188.2 (37.3)	< 0.001#	195.2 (38.6)	199.1 (39.2)	190.7 (37.6)	< 0.001#
Triglycerides, mg/dl, median (IQR)	104.4 (74.3, 152.2)	110.6 (78.8, 156.6)	96.5 (69.0, 145.1)	< 0.001¶	118.7 (80.6, 183.3)	120.9 (83.3, 181.6)	116.0 (77.9, 186.9)	0.091¶
LDL cholesterol, mg/dl, mean (SD)	116.2 (33.8)	119.9 (33.9)	112.1 (33.3)	< 0.001#	120.7 (36.3)	124.7 (36.5)	116.2 (35.5)	< 0.001#
HDL cholesterol, mg/dl, mean (SD)	51.5 (15.2)	51.9 (14.3)	51.1 (16.2)	0.013#	56.2 (20.4)	57.1 (19.0)	55.1 (21.9)	< 0.001#
Count of MS criteria (other than WC), n (%)				< 0.001*				0.003*
0	1198 (16.0)	570 (14.5)	628 (17.7)		1103 (23.0)	582 (22.7)	521 (23.4)	
1	2258 (30.2)	1105 (28.1)	1153 (32.5)		1544 (32.2)	801 (31.3)	743 (33.3)	
2	2112 (28.2)	1072 (27.3)	1040 (29.3)		1203 (25.1)	625 (24.4)	578 (25.9)	
3 or 4	1917 (25.6)	1186 (30.2)	731 (20.6)		938 (19.6)	552 (21.6)	386 (17.3)	

*BP* Blood pressure, *CHARLS* China Health and Retirement Longitudinal Study, *CHNS* China Health and Nutrition Survey, *MS* Metabolic syndrome, *NA* Not available, *LDL* Low density lipoprotein, *HDL* High density lipoprotein, *SD* Standard deviation, *IQ*R Interquartile range, *WC* Waist circumference

*χ^2^ test

# Two sample t test

¶ Mann-Whitney U test

### GAMs analysis

**Fig. 2:**
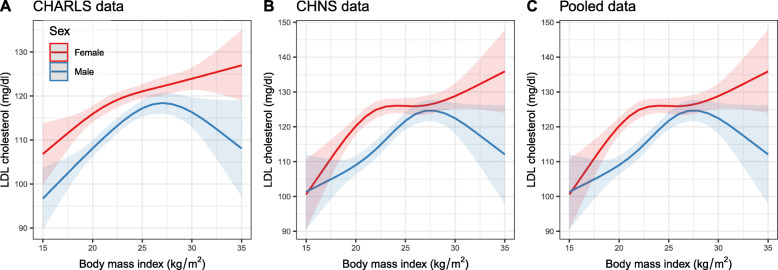
The nonlinear relationship between BMI and LDL-C in females and males using generalized additive models with the factor-smooth interaction terms for sex*BMI

Table 2 shows the estimated regression coefficients from GAMs with factor-smooth interaction terms for sex*BMI. The interactions term sex*BMI, sex, age, residence, education, and cohort were significantly associated with LDL-C in the pooled analysis. Estimated smooth functions plot shows the non-linear association between BMI and LDL-C in females and males (*P* < 0.001 for smooth terms) (Fig. 2**)**. The non-linear relationship between BMI and serum TC, HDL-C and TG by sexes is also observed (Supplementary Fig. 2).

**Table 2:** Estimated regression coefficients (*β*) from the generalized additive model with a factor-smooth interaction

	CHARLS (*n* = 7485)		CHNS (*n* = 4788)		Pooled (*n* = 12,273)	
	*β (SE)*	*P* value	*β (SE)*	*P* value	*β (SE)*	*P* value
Intercept	100.71 (3.27)	< 0.001	107.32 (4.53)	< 0.001	105.12 (2.7)	< 0.001
Age						
Per 1 year	0.34 (0.05)	< 0.001	0.35 (0.07)	< 0.001	0.36 (0.04)	< 0.001
Sex						
Female	Reference		Reference		Reference	
Male	−6.13 (1.05)	< 0.001	−7.47 (1.39)	< 0.001	−6.67 (0.84)	< 0.001
Sex*BMI (smooth)						
Female	See Fig. 2 (A)	< 0.001	See Fig. 2 (B)	< 0.001	See Fig. 2 (C)	< 0.001
Male	See Fig. 2 (A)	< 0.001	See Fig. 2 (B)	< 0.001	See Fig. 2 (C)	< 0.001
Residence						
Urban	Reference		Reference		Reference	
Rural	□−0.27 (0.83)	0.750	□−4.76 (1.18)	< 0.001	□−1.88 (0.68)	0.006
Education						
Below high school	Reference		Reference		Reference	
High school or above	0.33 (1.32)	0.805	1.27 (1.39)	0.358	1.26 (0.94)	0.180
Marital status						
Others	Reference		Reference		Reference	
Married	□−0.56 (1.32)	0.617	0.22 (1.65)	0.892	□−0.36 (0.93)	0.702
Smoking						
No	Reference		Reference		Reference	
Yes	□−1.11 (1.06)	0.294	□−0.48 (1.42)	0.734	□−0.8 (0.85)	0.343
Drinking						
No	Reference		Reference		Reference	
Yes	□−1.22 (1.32)	0.188	□−0.78 (1.34)	0.562	□− 1.08 (0.77)	0.158
Cohort						
CHNS					Reference	
CHARLS					3.90 (0.64)	< 0.001
	R-sq.(adj) = 0.0299		R-sq.(adj) = 0.0419		R-sq.(adj) = 0.0381

### Threshold analysis

Table 3 shows the findings from generalized linear models using linear splines. In females, BMI was linearly associated with LDL-C below a BMI of 20.33 kg/m^2^ (slope: 3.45, 95% CI: 1.40 to 5.49), and a gradual increase above beaks point (slope: 0.69, 95% CI: 0.34 to 0.96, *P* < 0.001 for slope differences) in pooled analysis (Model 1). In males, the association between BMI and LDL-C was inverse U-shaped, with a BMI break point of 27.1 kg/m^2^ (slope: 1.82, 95% CI: 1.44 to 2.21 for BMI < 27.1 kg/m^2^; slope: -1.54, 95% CI: − 2.97 to − 0.13 for BMI ≥ 27.1 kg/m^2^). The estimated beak points and slope between BMI and LDL-C did not significantly change with additionally adjustment for hypertension and diabetes (Model 2). The finding was robustness after adjustment with use of lipid-lowering (Model 2a) and menopause status (Model 2b) in CHARLS (Table 3). The threshold analysis also revealed that there was a cohort differences in the estimated beak points and slope between BMI and LDL-C (Table 3).

**Table 3:** Slopes (95% CIs) of the association of BMI with LDL cholesterol among individuals below or above the breakpoint by sex in the CHALRS and CHNS

Population	BMI (kg/m^2^)	LDL cholesterol slope (mg/dL per kg/m^2^)		*P* Value for differences*
	Estimated Breakpoint (95% CI)	< Breakpoint (95% CI)	≥ Breakpoint (95% CI)	
Female				
CHARLS (*n* = 3933)				
Model 1	20.49 (18.10 to 22.89)	2.67 (0.51 to 4.84)	0.72 (0.34 to 1.11)	0.023
Model 2	20.51 (18.09 to 22.92)	2.73 (0.55 to 4.92)	0.77 (0.38 to 1.17)	0.022
Model 2a	20.52 (18.04 to 22.99)	2.72 (0.50 to 4.94)	0.77 (0.38 to 1.17)	0.022
Model 2b	20.51 (17.99 to 23.02)	2.67 (0.48 to 4.86)	0.79 (0.39 to 1.19)	0.028
CHNS (*n* = 2560)				
Model 1	20.11 (18.56 to 21.66)	5.30 (1.31 to 9.28)	0.58 (0.07 to 1.10)	0.001
Model 2	20.14 (18.61 to 21.67)	5.26 (1.39 to 9.13)	0.60 (0.07 to 1.13)	0.001
Pooled (*n* = 6493)				
Model 1	20.33 (18.84 to 21.83)	3.45 (1.40 to 5.49)	0.65 (0.34 to 0.96)	< 0.001
Model 2	20.34 (18.92 to 21.75)	3.49 (1.54 to 5.45)	0.69 (0.37 to 1.00)	< 0.001
Male				
CHARLS (*n* = 3552)				
Model 1	25.87 (24.23 to 27.51)	1.74 (1.19 to 2.30)	−1.06 (−2.35 to 0.23)	< 0.001
Model 2	25.87 (24.22 to 27.52)	1.74 (1.18 to 2.30)	−1.05 (−2.35 to 0.24)	< 0.001
Model 2a	25.87 (24.34 to 27.50)	1.74 (1.17 to 2.30)	−1.07 (− 2.37 to 0.23)	< 0.001
CHNS (*n* = 2228)				
Model 1	27.27 (25.31 to 29.23)	2.23 (1.60 to 2.86)	−1.47 (−4.00 to 1.06)	0.001
Model 2	27.27 (25.29 to 29.26)	2.27 (1.63 to 2.91)	−1.38 (−3.91 to 1.15)	0.001
Pooled (*n* = 5780)				
Model 1	27.06 (25.78 to 28.34)	1.82 (1.44 to 2.21)	−1.54 (−2.97 to −0.13)	< 0.001
Model 2	27.06 (25.78 to 28.34)	1.84 (1.45 to 2.31)	−1.50 (−2.92 to −0.09)	< 0.001

Model 1: Adjusted for age, residence, education levels, marital status, drinking, smoking status, and cohort (when pooled analysis)

Model 2: Adjusted for covariates in Model 1 plus hypertension and diabetes

Model 2a: Adjusted for covariates in Model 1 plus hypertension, diabetes and use of lipid-lowering

Model 2b: Adjusted for covariates in Model 1 plus hypertension, diabetes, use of lipid-lowering and menopause status

*P for slope differences between lower and upper anthropometric ranges

### Subgroup analysis

**Fig. 3:**
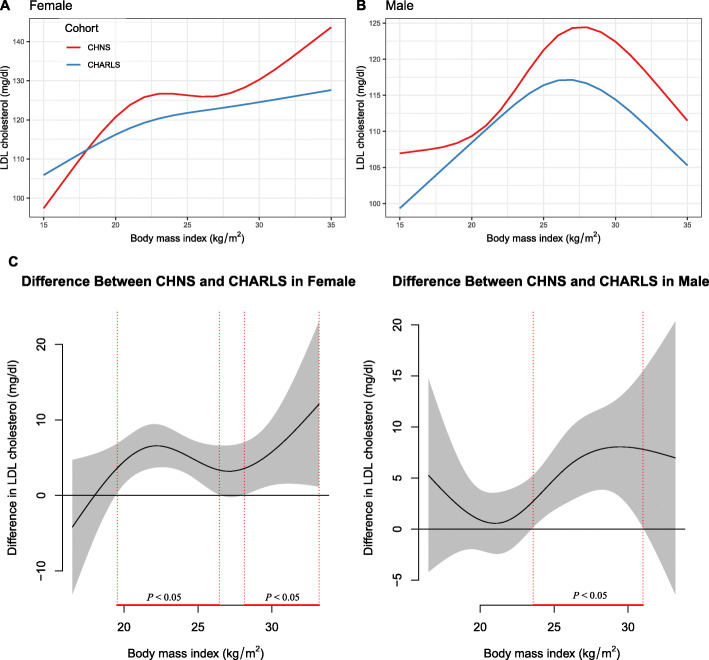
The nonlinear relationship between BMI and LDL-C in females and males using generalized additive models with the factor-smooth interaction terms for sex*BMI, by cohort

**Fig. 4:**
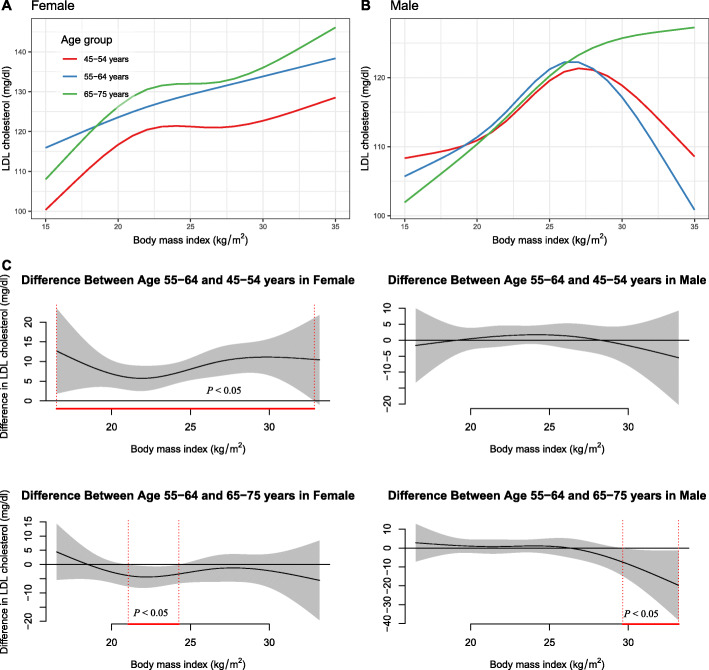
The nonlinear relationship between BMI and LDL-C in females and males using generalized additive models with the factor-smooth interaction terms for sex*BMI, by age groups

**Fig. 5:**
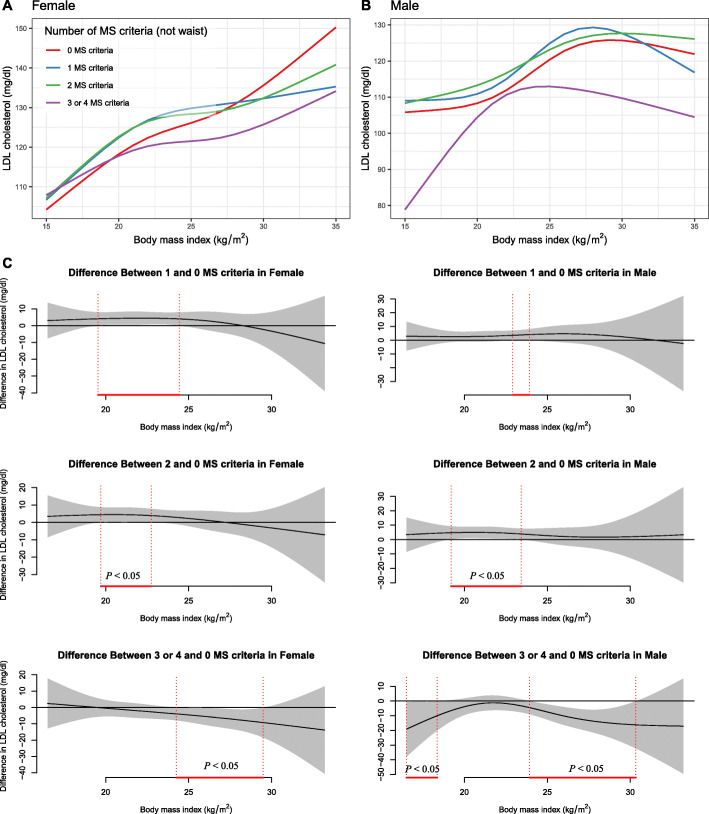
The nonlinear relationship between BMI and LDL-C in females and males using generalized additive models with the factor-smooth interaction terms for sex*BMI, by the number of metabolic syndrome criteria (without waist circumference)

The non-linear association between BMI and LDL-C in males and females was varied by cohort source, age groups, and the number of MS criteria (Fig. 3, Fig. 4, and Fig. 5).

## Discussion

In this study, we found that in the population with normal or light weight, the association between BMI and LDL-C was linearly and positively correlated, while in the overweight population, with increasing BMI, LDL-C levels gradually tended to be flat or even decreased. The trend of the above association was different between sexes, with an inverted U-shaped association in males.

Evaluating the association between BMI and CVD risk factors (i.e., LDL-C) will help prevent the occurrence and development of diseases while elucidation of the non-linear relationship between them is conducive to quantifying risk levels for people with different BMI levels. Studies have explored the linear or non-linear association between BMI and CVD risk factors, as well as differences in above associations between sexes and age subgroups [20–22]. For example, a study based on a Chinese cohort population revealed that high BMI is an indicator for increased risk of arterial stiffening during adulthood in males. However, this association was not established in females [21]. An American study found a U-shaped association between BMI and mortality [22].

The association between BMI and lipid metabolites was previously assessed by HDL-C or TG levels and found to be linear [23, 24], however, the dose-response relationship between BMI and LDL-C has not been conclusively determined. Several studies have reported a positive association between BMI and LDL-C [25–28], whereas other studies reported contrasting findings [29, 30]. A study performed using two cross-sectional nationally representative data from the U.S. and Spain found an inverted U-shaped association between BMI and LDL-C [15], consistent with a similar study involving with non-diabetic American Indians [30]. In addition, the association between BMI and LDL-C has been shown to differ between sex and age subgroups [28, 31]. Although the above studies revealed the non-linear association between BMI and LDL-C, they are limited to the European and American population, and most of the studies only explored the single sex. In the Chinese population, the role of serum LDL-C in different BMI levels in the elderly population remains unclear [32]. Our findings show the benefits of weight loss for people with different BMI levels in reducing the risk of occurrence of CVD.

We found that the association between BMI and LDL-C exhibits as an inverted U-shape in males, consistent with previous studies involving with U.S. and Spain populations [15, 30]. In non-obese people, TG rich very low-density lipoprotein (VLDL) were converted into cholesterol rich intermediate density lipoprotein and LDL for adipogenesis, leading to a positive correlation between BMI and LDL in lean individuals [15]. However, when lipid deposition in adipose tissue reached the maximum limit or other lipid metabolism disorders, TG may accumulated in VLDL, resulting in a decrease in LDL formation [33]. Another possible explanation is that adipose tissues store large amounts of cholesterol, thus buffering the cholesterol load of the liver [34]. Secretion of bile cholesterol in obese people increases with the accumulation of adipose cells [35]. Therefore, in obese people, normal LDL-C levels may suggest that increased adipocytes are maintaining cholesterol homeostasis. In addition, proprotein convertase subtilisin/kexin type 9 (PCSK9) is positively associated with BMI and LDL-C levels, which confirms the association between BMI and LDL-C [36, 37]. It has been reported that PCSK9 concentrations are correlated with age, gender, MS, and menopausal status, which may lead to alterations in the linear association [38]. Despite these findings, there is no direct evidence of the underlying mechanisms for these associations.

We also evaluated the association between BMI and LDL-C stratified by sexes. The inverted U-shaped associations are more pronounced in males than in females. Most studies on the above-mentioned associations involved in a single gender, especially in males [14, 26, 28, 29] . Moreover, after eliminating the confounding factor of body fat percentage, the associations between physical fitness levels and plasma lipid profiles between male and female genders were found to be different [39]. Sex-specific hormones can lead to sex differences in lipid metabolism [40]. LDL-C levels are correlated with menopausal status, that is, postmenopausal females have higher concentrations of LDL-C [41–43]. For example, PCSK9 concentrations were significantly higher in females than in males [38]. Thus, differences in hormonal status between males and females may lead to different associations between BMI and LDL-C. Some studies have shown that BMI and LDL-C exhibit a positive linear relationship in females [31]. Quantitatively, in a previous study, LDL-C levels among females with BMI between 27.1 kg/m^2^ and 30.0 kg/m^2^ increased by 17 mg/dl compared to those whose BMI was between 21.1 kg/m^2^ and 23.0 kg/m^2^ [31]. We found that LDL-C levels increased with BMI. Moreover, for the first time, we show that there were one turning point in the association between BMI and LDL-C, that is, the slope began to flatten near the edge of obesity, and gradually increased again when BMI reached 27.1 kg/m^2^. The sex-specific BMI and LDL-C associations may be due genetic vulnerability and hormonal status [24, 28, 29]. Females store more lipids and have higher percent body-fat, less visceral white adipose tissue, and more subcutaneous adipose tissue than males. Furthermore, females have a higher rate of TG synthesis compared to males [30]. Although we postulated that hormonal status and sex-specific effects in lipid metabolism may be responsible for above association, specific mechanisms have not been established.

We confirmed supported that MS status plays a role in association between BMI and LDL-C, that is, with aggravation of metabolic impairment, the turning point of the association curve between BMI and LDL-C gradually appeared earlier. In the extreme group with three or four MS components, the association trend in males was weakened, while it was more unstable in females, in tandem with findings from a previous US population study [15]. We further investigated dose-response association between BMI and LDL-C among males and females, respectively. MS, which are associated with increased BMI, are caused by abnormal functions of the adipose tissue. Another cause may be that, in the regulation of homeostasis mechanism, lipid transport cholesterol may reduce the risk of diabetes and lead to high levels of LDL-C. However, when the homeostatic mechanism breaks, abnormal cholesterol synthesis and transport may also break the linear relationship between BMI and LDL-C.

### Comparisons with previous studies

Previous study majorly involved European and American populations, exploring the linear relationship between BMI and LDL-C [15]. This study explored the non-linear association between BMI and LDL-C in the Asian population, and further explored the association between different genders and MS status subgroups. A clear dose-response for Asian populations by reducing body weight and reducing LDL-C levels were estimated.

### Study strengths and limitations

This study has several strengths. First, two large population-based nationally representative surveys in China were used to examine the association between BMI and LDL-C levels. Second, we addressed sex differences in the non-linear association between BMI and LDL-C by estimating the factor-smooth interaction between sex (factor) and BMI (smooth function) in the generalized additive models. Third, both datasets used standardized methods to collect exposure, outcome, and confounder. However, there are some limitations. First, this study is a cross-sectional study design, therefore, it did not establish the causal relationship between BMI and LDL-C. Thus, longitudinal studies should be performed to confirm the findings in this study using generalized additive mixed model. Second, the data of obesity-related indices, such as percent body fat and visceral fat area, was not available in the current study. Third, although the association between BMI and LDL-C was reevaluated by a series of sensitivity analyses, residual confounders still existed, such as hormone treatment. Lastly, cardiometabolic health was defined according to the severity of MS in the current analysis. Further prospective cohort studies are warranted to explore the role of cardiometabolic health on the association between BMI and LDL-C among males and females using more accurate quantitative indicators, such as coronary artery calcium score or carotid intima-media thickness.

## Conclusions

In people with normal or light weight, the association between BMI and LDL-C is linearly and positively correlated in both sexes, in the overweight people, with increasing BMI, the level of LDL-C tends to be flat or decreased in males. Future studies are warranted to determine the longitudinal association between BMI and LDL-C level.

## Supplementary Information

**Additional file 1: Table S1.** Baseline Characteristics by CHARLS and CHNS. **Figure S1.** The distribution of BMI and LDL-C levels by sexes and cohort. **Figure S2.** The association of BMI and TC, HDL-C, and TG levels by sexes.

## Data Availability

The original datasets are publicly available for CHARLS (http://charls.pku.edu.cn/) and CHNS (https://www.cpc.unc.edu/projects/china).
