# Supplementary material for: Sex differences in the non-linear association between BMI and LDL cholesterol in middle-aged and older adults: findings from two nationally representative surveys in China
Source: Lipids Health Dis. 2021 Nov 14;20:162. doi: 10.1186/s12944-021-01591-w (PMC8590757; doi:10.1186/s12944-021-01591-w)
Supplement: Supplementary file 1 — Additional file 1: Table S1. Baseline Characteristics by CHARLS and CHNS. Figure S1. The distribution of BMI and LDL-C levels by sexes and cohort. Figure S2. The association of BMI and TC, HDL-C, and TG levels by sexes. [file 12944_2021_1591_MOESM1_ESM.docx]

| Table S1. Baseline Characteristics by CHARLS and CHNS | | | |
| --- | --- | --- | --- |
|  | CHARLS (n = 7485) | CHNS (n = 4788) | *P* value |
| Female, n (%) | 3933 (52.5) | 2560 (53.5) | 0.318* |
| Age, years, mean (SD) | 58 (8) | 57 (8) | 0.229# |
| 45-54 | 2831 (37.8) | 1959 (40.9) |  |
| 55-64 | 3083 (41.2) | 1784 (37.3) |  |
| 65-75 | 1571 (21.0) | 1045 (21.8) |  |
| Rural residence, n (%) | 4924 (65.8) | 3274 (68.4) | 0.003* |
| High school or above, n (%) | 760 (10.2) | 988 (20.6) | <0.001* |
| Married, n (%) | 6405 (85.6) | 4224 (88.2) | <0.001* |
| Drinking, n (%) | 2617 (35.0) | 1585 (33.1) | 0.034* |
| Smoking, n (%) | 2393 (32.0) | 1396 (29.2) | <0.001* |
| BMI, kg/m^2^, mean (SD) | 23.4 (3.5) | 23.7 (3.3) | <0.001# |
| Underweight | 476 (6.4) | 242 (5.1) |  |
| Normal | 4000 (53.4) | 2451 (50.4) |  |
| Overweight | 2193 (29.3) | 1613 (33.7) |  |
| Obese | 816 (10.9) | 518 (10.8) |  |
| Hypertension, n (%) | 2914 (38.9) | 1859 (38.8) | 0.907* |
| Diabetes, n (%) | 1140 (15.2) | 463 (9.7) | <0.001* |
| Hypercholesterolemia, n (%) | 1072 (14.3) | 577 (12.1) | <0.001* |
| History of medication use, n (%) |  |  |  |
| Hypertension medications | 1163 (15.5) | 184 (3.8) | <0.001* |
| Diabetes medications | 233 (3.1) | 169 (3.5) | 0.206* |
| Lipid-lowering therapy | 287 (3.8) | NA |  |
| Menopause, n (%) | 2864 (72.8) | NA |  |
| Waist circumference, cm, mean (SD) | 83.9 (12.1) | 84.3 (9.9) | 0.085# |
| Systolic BP, mmHg, mean (SD) | 130 (21) | 129 (19) | 0.033# |
| Diastolic BP, mmHg, mean (SD) | 76 (12) | 82 (11) | <0.001# |
| Fasting glucose, mg/dl, mean (SD) | 109.5 (36.2) | 100.0 (28.6) | <0.001# |
| Total cholesterol, mg/dl, mean (SD) | 193.2 (37.9) | 195.2 (38.6) | 0.005# |
| Triglycerides, mg/dl, median (IQR) | 104.4 (74.3, 152.2) | 118.7 (80.6, 183.3) | <0.001¶ |
| LDL cholesterol, mg/dl, mean (SD) | 116.2 (33.8) | 120.7 (36.3) | <0.001* |
| HDL cholesterol, mg/dl, mean (SD) | 51.5 (15.2) | 56.2 (20.4) | <0.001* |
| Count of MS criteria (other than WC), n (%) |  |  | <0.001* |
| 0 | 1198 (16.0) | 1103 (23.0) |  |
| 1 | 2258 (30.2) | 1544 (32.2) |  |
| 2 | 2112 (28.2) | 1203 (25.1) |  |
| 3 or 4 | 1917 (25.6) | 938 (19.6) |  |
| BP=blood pressure, CHARLS=China Health and Retirement Longitudinal Study, CHNS=China Health and Nutrition Survey, MS=metabolic syndrome, NA=not available, LDL=low density lipoprotein, HDL=high density lipoprotein, SD=standard deviation, IQR=interquartile range, WC=waist circumference. | | | |
| * χ^2^ test | | | |
| # Two sample t test | | | |
| ¶ Mann-Whitney U test | | | |


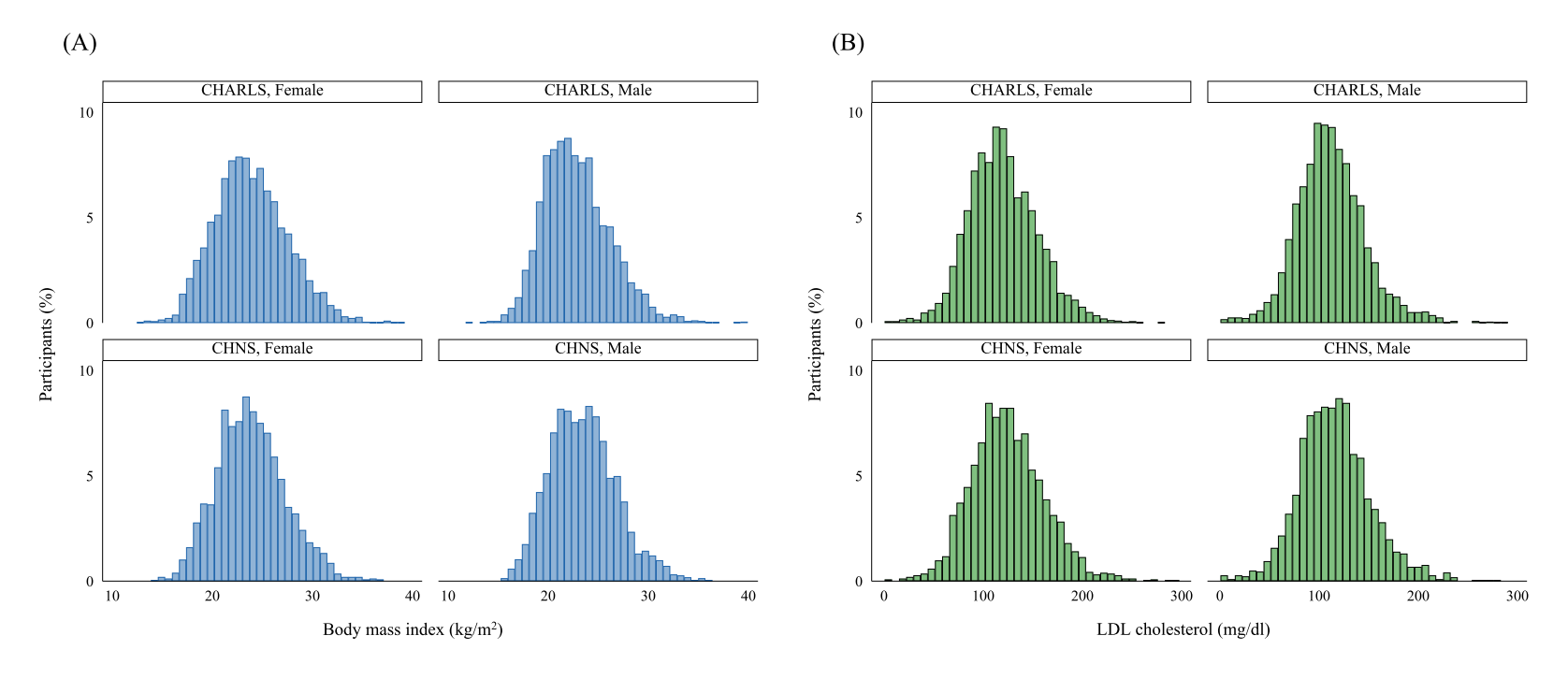


**Figure S1. The distribution of BMI and LDL-C levels by sexes and cohort.**


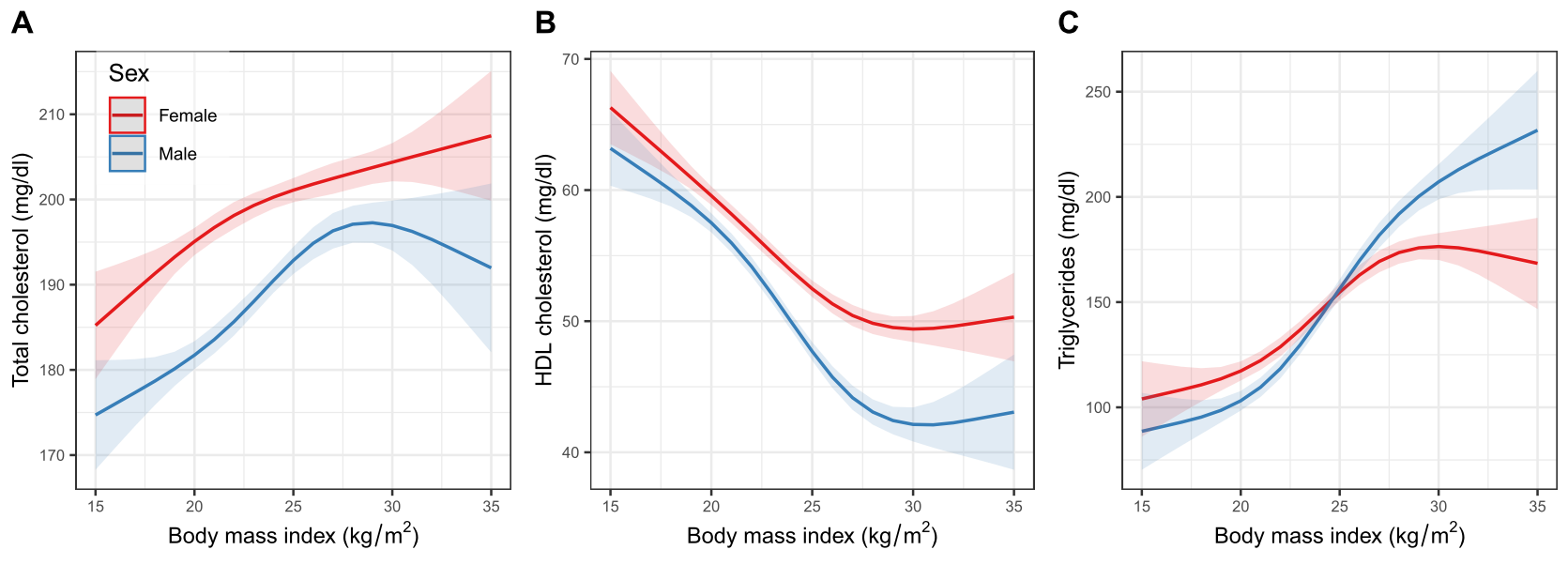


**Figure S2. The association of BMI and TC, HDL-C, and TG levels by sexes.**
